# Incidental melanoma and thyroid cancer lead to diagnosis of Lynch syndrome and endometrial cancer: A case report

**DOI:** 10.1016/j.jdcr.2024.06.023

**Published:** 2024-07-05

**Authors:** Molly E. Kuo, Emily H. Smith, Jaclyn Plotzke, May Chan, Tobias Else, Kelly B. Cha

**Affiliations:** a Medical Scientist Training Program, University of Michigan, Ann Arbor, Michigan; b Departments of Dermatology and Dermatopathology, Saint Louis University, St. Louis, Missouri; c Department of Dermatology, University of Michigan, Ann Arbor, Michigan; d Department of Pathology, University of Michigan, Ann Arbor, Michigan; e Division of Metabolism, Endocrinology and Diabetes, Department of Internal Medicine, University of Michigan, Ann Arbor, Michigan

**Keywords:** Lynch syndrome, melanoma, microsatellite instability, PMS2

## Introduction

Hereditary nonpolyposis colorectal cancer, or Lynch syndrome, is an autosomal dominant cancer predisposition syndrome caused by pathogenic germline variants in DNA mismatch repair genes (mutL homolog 1, mutS homolog 2, mutS homolog 6, PMS1 homolog 2 [*PMS2*]) or deletions in epithelial cell adhesion molecule.^1^^,^^2^ Lynch syndrome is most clearly associated with increased risk of colorectal and endometrial cancers, as well as skin cancers including sebaceous neoplasms and keratoacanthoma-type squamous cell carcinoma.^3^ Melanoma has also been reported.^4^ We report a case of a 47-year-old woman with history of papillary thyroid cancer whose diagnosis of melanoma led to cancer genetics evaluation revealing a pathogenic *PMS2* variant and subsequent identification of endometrial cancer.

## Case

A 47-year-old woman of German, Irish, and English ancestry and a history of atypical nevi presented to dermatology clinic for evaluation of a lesion on her left lower leg. The lesion had been present for decades, and over the preceding year, the center had become raised and traumatized while shaving. Her history was remarkable for papillary thyroid cancer with lymph node metastasis diagnosed at age 44 and treated with total thyroidectomy and modified radical lymph node dissection followed by adjuvant radioactive iodine. Her family history included a sister with basal cell carcinoma, father with prostate cancer, and paternal aunt and maternal grandmother with breast cancer.

Dermatologic physical examination revealed several banal and some slightly atypical-appearing brown macules and flat papules as well as a 5 mm light brown macule with central pink papule on the left lower leg. A shave biopsy was performed.

Histopathologic examination revealed a markedly atypical compound melanocytic proliferation, favoring melanoma, invasive to 1.1 mm Breslow depth, nonulcerated, with a mitotic rate of 1/mm^2^. Lesional cells overexpressed PRAME and demonstrated a B-Raf proto-oncogene, serine/threonine kinease c.1799T>A (p.V600E) mutation. BRCA1 associated protein 1 expression was retained. Molecular investigation revealed a telomerase reverse transcriptase promoter mutation further supporting a diagnosis of melanoma. She underwent wide local excision with 1 cm margins and sentinel lymph node biopsy of 1 left inguinal node, which was positive for melanoma (<1% of lymph node surface area). Staging imaging including computed tomographies of the chest, abdomen, pelvis, and left lower leg, magnetic resonance imaging brain, and positron emission tomography-computed tomography showed no evidence of distant metastases. For pathologic stage IIIA melanoma (T2a N1a M0), she is undergoing MSLT II-informed surveillance with regular imaging including ultrasound of the left inguinal basin.

Given her personal history of multiple cancers and family history of cancers, she was referred to cancer genetics and pursued genetic testing with a 91-gene panel (Ambry Laboratories), demonstrating a germline *PMS2* pathogenic variant (EX8del) consistent with Lynch syndrome. She reported no concerning gastrointestinal symptoms, and baseline colonoscopy and upper gastrointestinal endoscopy were unremarkable. Notably, she had a multiyear history of abnormal uterine bleeding attributed to adenomyosis. A prior endometrial biopsy was negative for malignancy. In light of her newly-identified genetic risk, she was re-evaluated by obstetrics and gynecology who recommended risk-reducing total hysterectomy and bilateral oophorectomy, with the secondary goal of relieving her abnormal uterine bleeding. Histopathological examination of the resection specimen revealed endometrial endometrioid adenocarcinoma with mucinous features. Current surveillance includes close follow-up by dermatology, oncology, endocrinology, and gynecology.

**Fig 1:**
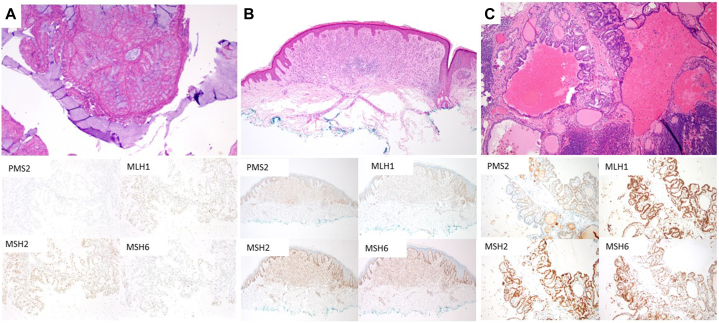
Immunohistochemical staining pattern observed in patient’s surgical specimens. **A,** Endometrial adenocarcinoma displaying loss of PMS2 and retention of MLH1, MSH2, and MSH6. **A,** Hematoxylin and eosin, 40× original magnification; immunohistochemical stains 100× original magnification. **B,** Melanoma showing retention of all MMR markers. **C,** Lymph node containing metastatic papillary thyroid carcinoma showing retention of all MMR markers. **B** and **C,** Hematoxylin and eosin, 40× original magnification; immunohistochemical stains 40× original magnification.

Impaired function of mismatch repair proteins in Lynch syndrome is associated with high microsatellite instability.^5^^,^^6^ To query the relationship of thyroid cancer, melanoma, and endometrial cancer to this patient’s *PMS2* variant, microsatellite instability testing was performed. DNA was extracted from tumor tissue and corresponding normal tissue and subjected to multiplex polymerase chain reactions amplifying 8 microsatellite markers. The resulting products were compared to determine if novel microsatellite lengths existed in the tumor, indicating instability. Her endometrial cancer had high microsatellite instability (novel microsatellite lengths were present in 5/8 markers in the tumor tissue) while her thyroid cancer and melanoma had low microsatellite instability (0/8 markers demonstrated microsatellite instability). Immunohistochemical evaluation revealed isolated loss of *PMS2* in her endometrial carcinoma, with retention of all mismatch repair genes in her thyroid and melanoma specimens (Fig 1).

## Discussion

A new diagnosis of melanoma in a 47-year-old patient with a history of thyroid cancer and family history of breast and prostate cancer prompted referral to cancer genetics and subsequent diagnosis of Lynch syndrome and endometrial cancer. Evaluation of microsatellite instability in each tumor suggests that melanoma and thyroid cancer were incidental to, and endometrial cancer was associated with, the identified variant in *PMS2*.

An estimated 7% to 15% of melanomas are diagnosed in individuals with a family history of melanoma, and some of these individuals have an identifiable germline variant in a tumor predisposition gene.^7^ Findings suggesting inherited cancer predisposition include early age of onset, multiple primary cancers, and relevant family history.^8^ In our patient, referral was offered primarily because she had been diagnosed with 2 primary cancers in her 40s, and this was considered potentially suggestive of inherited risk. Pathogenic genetic variants in several genes are associated with increased melanoma risk. Mutations in cyclin dependent kinase inhibitor 2A are the most common cause of hereditary melanoma.^7^ Variants in several other genes, including BRCA1-associated protein 1, protection of telomeres protein 1, and telomerase reverse transcriptase, are also associated with increased risk for melanocytic tumors. Melanoma risk is also thought to be increased in cancer predisposition syndromes including Cowden syndrome, Li-Fraumeni syndrome, and hereditary breast and ovarian cancer syndrome, caused by pathogenic variants in phosphatase and tensin homolog, tumor protein 53, and *BRCA1* and *BRCA2*, respectively.^8^ A diagnosis of melanoma should prompt dermatologists to elicit a personal and family history of cancer to screen for hereditary cancer syndromes.^8^^,^^9^ Management of melanoma is typically unchanged by the identification of a cancer predisposition syndrome, but such a diagnosis allows appropriate counseling and surveillance for other cancers, as well as identification of other at-risk family members.

Many inherited cancer predisposition syndromes have clear associations with a defined subset of cancers and tentative associations with others. When common cancers such as melanoma occur in patients with diagnosed cancer predisposition syndromes, it can be difficult to determine if the diagnosis is incidental or related to the underlying pathogenic variant. In Lynch syndrome, pathogenic variants cause high microsatellite instability, which is evident on histopathology with appropriate testing. In our patient, we were able to illustrate that her melanoma and thyroid cancer, though relevant in prompting genetics referral, were ultimately incidental to her diagnosis of Lynch syndrome. This serves as a reminder that even in patients with cancer predisposition syndromes, an individual cancer diagnosis, particularly a relatively common cancer diagnosis and/or a diagnosis outside the core known manifestations of the syndrome, should not be assumed to be directly related to the pathogenic variant. Clinicopathologic studies, however, can offer support or refutation of a biologic connection and contribute to refinement over time of appropriate screening recommendations for associated cancer diagnoses.

## Conflicts of interest

None disclosed.
